# Pharmacogenomics, Pharmacokinetics and Circulating Proteins As Biomarkers for Bevacizumab Treatment Optimization in Patients with Cancer: A Review

**DOI:** 10.3390/jpm10030079

**Published:** 2020-08-04

**Authors:** Apostolos Papachristos, Gregory B. Sivolapenko

**Affiliations:** Laboratory of Pharmacokinetics, Department of Pharmacy, School of Health Sciences, University of Patras, 26504 Patras, Greece; gsivolap@upatras.gr

**Keywords:** bevacizumab, pharmacogenomics, pharmacokinetics, anti-angiogenetic therapy, biomarkers, therapeutic drug monitoring, VEGF-A, precision medicine

## Abstract

Bevacizumab is a monoclonal antibody that targets VEGF-A and inhibits tumor angiogenesis. Bevacizumab is approved for the treatment of various cancer, including metastatic colorectal cancer (mCRC), ovarian cancer, lung cancer, and others. Thus, it is widely used in oncology, but contrary to other therapeutic classes, there is still a lack of validating predictive factors for treatment outcomes with these agents. In recent years, the research for factors predictive of anti-VEGF treatments and especially bevacizumab response has been one of the most competitive translational research fields. Herein, we review and present the available literature of the clinical use of biomarkers, pharmacogenomics (PG), and therapeutic drug monitoring (TDM) approaches that can be used for the optimization of bevacizumab use in the era of precision medicine.

## 1. Introduction

In 1971, Judah Folkman described first the process of angiogenesis and its contribution to tumor growth [1]. Since then, angiogenesis pathway has been extensively studied. The dominant factor controlling angiogenesis is a glycoprotein called vascular endothelial growth factor (VEGF) [2,3,4]. Several studies have highlighted the prognostic and predictive significance of VEGF and VEGFR in colorectal [5,6], lung [7,8], gastric [9], and pancreatic cancers [10]. Thus, angiogenesis and especially VEGF-A have become attractive pharmacologic targets. Bevacizumab, a recombinant humanized IgG1 monoclonal antibody, binds to circulating VEGF-A and the blocking of VEGF-A, which results in the inhibition of tumor angiogenesis, growth, and metastases [11].

In Europe and the USA, bevacizumab has been approved as monotherapy or in combination with chemotherapy and in various dosage regimens for the treatment of metastatic colorectal cancer (mCRC), metastatic breast cancer (mBC), unresectable advanced, metastatic or recurrent non-small cell lung cancer (NSCLC), advanced or metastatic renal-cell cancer (RCC), advanced, recurrent glioblastoma, advanced ovarian, and cervical cancers [12,13].

Most of these approvals are based on improvements in progression-free survival (PFS), not overall survival (OS) [14,15]. Firstly, in mBC, all five major randomized phase III trials (AVF2119g, E2100, AVADO, RIBBON-1, and RIBBON-2) revealed no significant increase in OS [16]. Similarly, phase III trials did not find any significant benefit in ovarian cancer (GOG-0218, ICON7, AURELIA, and OCEANS), metastatic RCC (AVOREN and CALGB 90206), glioblastoma (AVAglio and TROG 0825), NSCLC (AVAiL and BeTa) [14]. Moreover, no OS benefit was observed in five of seven mCRC trials (NO16966, NSABP C-08, ML18147, and MAX) [14]. However, the product continues to be widely used. Since its first approval in 2004, bevacizumab has generated sales of more than $50 billion in drug costs alone, which represents a financial burden on healthcare systems worldwide [14,15].

Besides, contrary to other therapeutic classes in oncology, there is still a lack of validating predictive factors for treatment outcomes with bevacizumab. The ability to use therapy towards well-selected subgroups of patients would increase the likelihood of benefits and would improve cost-effectiveness and therapeutic outcomes. In recent years the research for factors predictive of anti-VEGF treatments and especially bevacizumab response has been one of the most competitive translational research fields. Herein, we review the available literature and further delve into the identification, and the clinical use of proteinic and genetic-pharmacogenomic biomarkers, as well as therapeutic drug monitoring (TDM) approaches that can be used for the optimization of bevacizumab use in the era of precision medicine.

## 2. Proteinic Biomarkers

The measurement of concentrations of circulating proteins is an attractive biomarker strategy, as blood is easily accessible, the assays are inexpensive, and the proteins may be readily and quantitatively measured by automated methods such as enzyme-linked immunosorbent assays (ELISA). Proteins have been assessed as potential predictive biomarkers in mBC, mCRC, lung cancer, ovarian cancer, RCC, and glioblastoma.

### 2.1. Metastatic Breast Cancer (mBC)

Burstein HJ et al. sought to determine the efficacy and safety of bevacizumab and vinorelbine and to explore the role of baseline plasma VEGF-A as a predictor of treatment outcome. Levels > 32.6 pg/mL were significantly associated with a shorter PFS (*p* = 0.003) [17]. AVF2119g trial the effect of capecitabine or capecitabine plus bevacizumab. Primary tissue samples were examined for biomarkers discovery. The panel included VEGF-A, VEGF-B, thrombospondin-2 (THBS-2), Flt4, VEGF-C, Platelet-derived growth factor C (PDGF-C), neuropilin-1, delta-like ligand D114, Bv8, p53, and thymidine phosphorylase. Of them, VEGF-A expression showed a predictive significance (*p* = 0.01) for improved PFS when bevacizumab was added [18] (Table 1).

### 2.2. Metastatic Colorectal Cancer (mCRC)

A pivotal study in 813 patients, who received a combination of IFL and bevacizumab or placebo, assessed the expression of the stromal and epithelial VEGF-A, THBS-2 score, and microvessel density (MVD) in tissue samples. No biomarker associated with survival [19]. Plasma samples from HORIZON III (mFOLFOX6 plus cediranib or bevacizumab) trial were evaluated for baseline levels of VEGF-A, soluble VEGFR-2, and carcinoembryonic antigen (CEA). High baseline VEGF-A (>98 pg/mL) and CEA levels were associated with shorter PFS and OS regardless of treatment. In patients receiving bevacizumab, high expression of sVEGFR-2 was associated with increased PFS and OS [20]. In 2015, a study compared pre-and post-therapeutic VEGF-A IHC expression in 57 mCRC patients treated with bevacizumab and FOLFIRI in order to identify its potential role as predictive biomarkers. Low post-therapeutic VEGF-A scores (0 and 1) and decreased peri-therapeutic VEGF-A scores were significant predictive factors of response (*p* < 0.001). On the other hand, high pre-therapeutic VEGF-A scores (2 and 3) were not a significant predictive factor (*p* = 0.772). Furthermore, a decrease from score 2 to 1 or 0 between pre-treatment and post-treatment period was a significant predictor factor of response to bevacizumab (*p* < 0.001). Decreased peri-therapeutic VEGF-A scores were also significantly associated with higher 6-month PFS (*p* = 0.033), as well as with longer but not statistically significant OS (*p* = 0.094) [21]. Another group assessed several biomarkers at baseline, 3 and 12 days after a dose of bevacizumab monotherapy, 32 days after initiation of neoadjuvant bevacizumab, fluorouracil, and radiotherapy and 1 week before surgery (8 to 9 weeks after completion of preoperative treatment). Notably, patients who experienced greater than 2-fold increases in plasma PIGF after bevacizumab monotherapy had a minimal disease at surgery (*p* < 0.05). Furthermore, bevacizumab alone or in combination with chemoradiotherapy increased plasma PIGF, VEGF-A and soluble VEGFR (*p* < 0.0001). Thus, the researchers concluded that mainly PIGF and VEGF-A might serve as generic pharmacodynamics biomarkers for anti-VEGF therapy as the chemoradiotherapy alone did not seem to change VEGF-A or PlGF [22]. Finally, a meta-analysis that included 11 eligible studies regarding the association of baseline VEGF-A plasma or intratumoral levels with PFS, OS, and objective response, concluded that high levels could predict poor treatment effect of bevacizumab and chemotherapy in mCRC for both PFS (*p* = 0.0001) and OS (*p* < 0.0001) [23].

Beyond VEGF-dependent pathways, several groups have studied other angiogenesis biomarkers. Koeptz S et al. published their results in 2010, investigating whether the changes of 37 plasma cytokines and angiogenic factors can be potential markers of response or resistance to anti-VEGF treatment. Factors were measured with multiplex-bed and ELISA assays at baseline, during treatment and at the time of progression in 43 previously untreated patients, who received bevacizumab and FOLFIRI for mCRC. Significant elevation of pro-angiogenic cytokines, basic fibroblast growth factor (bFGF) (*p* = 0.046), PIGF (*p* < 0.001), and hepatocytes growth factor was observed before radiographic evidence of progressive disease. Based on this observation, investigators concluded that these elevations might represent mechanisms of resistance [24]. Rozoni M et al. measured with flow cytometry the absolute number of total circulating endothelial cells (tCECs), resting CECs (rCECs), and endothelial progenitor cells (CEPs) at baseline and before the administration of the third and sixth course of treatment in 40 mCRC patients treated with bevacizumab and chemotherapy combination (FOLFIRI, FOLFOX4, XELOX, FOLFOXIRI). They also compared their results with a control group of 50 healthy volunteers. Interestingly, when the absolute number of tCEC (*p* = 0.01) and rCEC (*p* = 0.007) was <40 cells/mL at baseline the patients showed longer PFS. Thus, they concluded that CECs could be useful biomarkers for response prediction [25]. Finally, a group from Japan collected blood samples from 99 mCRC patients treated with first-line bevacizumab and mFOLFOX6 or XELOX in order to measure intercellular adhesion molecule 1 (ICAM-1) and interleukin 8 (IL-8) plasma levels. High plasma levels of ICAM-1 were significantly associated with shorter PFS (*p* = 0.003) and high plasma levels of IL-8 with shorter PFS (*p* = 0.048) and OS (*p* = 0.002) [26] (Table 1).

Therefore, VEGF-A levels found to be a significant predictive biomarker in mCRC, too and other pathways have also been identified as promising.

### 2.3. Lung Cancer

During the E4599 phase II/III study, 878 NSCLC patients were randomized to receive carboplatin and paclitaxel with or without bevacizumab. Based on the fact that VEGF-A, bFGF, soluble ICAM-1, and E-selectin are increased in several tumors, researchers performed a prospective biomarker assessment and their correlation to treatment outcomes. Plasma levels were measured before first cycle and after cycle 2. Patients with high baseline VEGF-A levels (>35.7 pg/mL) had increased probability of a response if bevacizumab was added to their treatment regimen (33% vs. 7.7%, *p* = 0.01). In patients with baseline VEGF-A ≤35.7 pg/mL, the response was similar, 28.6% and 29% for bevacizumab/chemotherapy and chemotherapy only arm, respectively. Low VEGF-A levels were also significantly associated with PFS (6 vs. 4.5 months, *p* = 0.04). While VEGF-A was predictive of response, low ICAM-1 levels (≤260.5 ng/mL) were prognostic for survival (*p* = 0.00005) and predictive of response to treatment (32% vs. 14%, *p* = 0.02) and 1-year survival (65% vs. 25%) [27]. ABIGAIL was another phase II, open-label, randomized, international, and multicenter study, which investigated the correlation between biomarkers (VEGF-A, VEGFR-1, VEGFR-2, bFGF, E-selectin, ICAM-1, and PIGF) in plasma samples at baseline and through treatment with the response to bevacizumab. In total, 303 chemo naïve NSCLC patients were randomized to receive bevacizumab 7.5 mg/kg (154 patients) or 15 mg/kg (149 patients) in combination with chemotherapy (carboplatin and gemcitabine or carboplatin and paclitaxel). Primary specimen tumor samples were also analyzed for VEGF-A, VEGFR-1, VEGFR-2, bFGF, E-selectin, PIGF, neuropilin (NRP), ICAM-1 and CD31. Baseline and dynamic changes in plasma levels of VEGFR-1, VEGFR-2, bFGF, E-selectin, ICAM-1, and PIGF did not correlate with response to bevacizumab. However, low baseline plasma VEGF-A levels were correlated with longer PFS (7.4 vs. 6.1 months, *p* = 0.002) and longer median OS (19.8 vs. 11.1 months, *p* = 0.004). As a result, the authors reported that VEGF-A might be promising biomarker [28].

A phase II trial in patients with extensive-stage small-cell lung cancer assessed efficacy and safety of bevacizumab, cisplatin, and etoposide combination in 63 patients. Correlative studies were performed in order to explore any potential relationship between treatment outcome and plasma levels of VEGF-A, soluble vascular cell adhesion molecules (VCAM), ICAM-1, E-selectin, and bFGF. Blood samples collected before cycle 1 and after completion of cycle 2. High baseline VCAM levels predicted survival as they were associated with higher risk of progression (*p* = 0.05) and death (*p* = 0.01) compared to low levels. High bFGF and ICAM-1 levels also showed a trend toward a higher risk of death (*p* = 0.06 both). The response was not associated significantly with baseline VEGF-A (*p* = 0.43) or any other biomarker [29].

In agreement with other tumor types, VEGF-A, and ICAM-1 levels found to be promising predictive biomarkers.

### 2.4. Ovarian Cancer

In the ovarian cancer setting, phase III GOG-0218 trial, compared carboplatin and paclitaxel with placebo, bevacizumab followed by placebo, or bevacizumab followed by bevacizumab. Baseline plasma samples were available from 751 participants and were analyzed via multiplex ELISA technology for seven potential biomarkers (IL-6, Ang-2, osteopontin (OPN), stromal cell-derived factor-1 (SDF-1), VEGF-D, IL6 receptor (IL-6 R), and GP130). In patients treated with bevacizumab, high IL-6 levels found to be predictive of PFS (*p* = 0.007) and OS (*p* = 0.003) [30].

Levels of IL-6 were found to be significantly associated with clinical outcomes in patients treated with bevacizumab for ovarian cancer. Other promising biomarkers such as VEGF-A, ICAM-1, and IL-8 levels were not assessed in this setting.

### 2.5. Renal-Cell Cancer (RCC)

Baseline plasma samples were collected from 424 consenting patients with renal-cell cancer, participating at CALGB 90206 phase III trial that was comparing bevacizumab and interferon-A versus interferon alone. ELISA was used to measure 32 candidate factors related to tumor growth, angiogenesis, and inflammation. IL-6 and hepatocyte growth factor (HGF) levels were positively correlated with OS in patients treated with bevacizumab. The authors reported that these factors might identify patients who will benefit most from bevacizumab and could guide clinical decisions and patients selection in future trials [31].

In line with results from ovarian cancer, IL-6 seems to be a promising candidate biomarker.

### 2.6. Glioblastoma

Sathornsumetee S et al. used tumor specimens collected at diagnosis from 45 patients (27 glioblastoma multiforme and 18 anaplastic astrocytoma), who were treated with bevacizumab and irinotecan. They retrospectively evaluated tumor vascularity and expression of components of VEGF pathway and hypoxic response as predictive markers for radiographic response and survival in tumor expression of VEGF-A, VEGFR-2, CD31 hypoxia-inducible cardonic anhydrase 9 (CA9), and hypoxia-inducible factor-2a (HIF-2a) were semi quantitatively assessed by immunohistochemistry (IHC). High VEGF-A expression (mean positive area>5000 pixels/400 field) was associated with increased likelihood of radiographic response (*p* = 0.024). Moreover, high CA9 expression (mean positive area > 10,000 pixels/high-powered field) was associated with poor 1-year survival after initiation of bevacizumab treatment (37 vs. 74 weeks, *p* = 0.02). Similarly, a trend was observed between HIF-2a expression and poor 1-year survival (*p* = 0.07). No significant differences in radiographic response or survival were reported for VEGFR-2 and CD31 (*p* > 0.1) [32].

To sum up, there are enough data from various types of tumors to support the rationale for the use of specific biomarkers as prognostic or predictive factors for the response. The most promising of them seems to be plasma VEGF-A and ICAM-1 levels as it has been associated in many studies with the response, OS, and PFS. It is also feasible to measure it, in contrast to intra-tumoral factors, and there are commercially available and validated ELISA kits for them.

## 3. Genetic Biomarkers-Pharmacogenomics (PG)

Several research groups have investigated the role of specific genotypes and single nucleotide polymorphisms (SNPs) in outcomes of bevacizumab treatment, as there is a substantial germline genetic variability within angiogenesis pathway genes, which causes interindividual differences in angiogenic capacity and resistance to anti-angiogenesis treatment. Some studies have also investigated the effect of genetic polymorphisms in bevacizumab-induced toxicity Pharmacogenomic biomarkers have been assessed as potential predictive biomarkers in mBC, mCRC, lung cancer, ovarian, and glioblastoma.

### 3.1. Metastatic Breast Cancer (mBC)

The association of *VEGF-A* and *VEGFR-2* genotypes with clinical outcomes was evaluated in phase III E2100 study comparing paclitaxel with bevacizumab and paclitaxel as initial chemotherapy for mBC. DNA was extracted from 363 tumor block samples. In the paclitaxel arm, there was no significant association between any genotype and response rate (RR), PFS, or OS. OS was 25.2 months for paclitaxel arm and 26.7 months for the combination arm not subdivided by genotype (*p* = 0.16). However, the median OS was significantly longer for subgroups *VEGF-A* rs699947 (*p* = 0.035) and *VEGF-A* rs1570360 A/A (*p* = 0.047) in the combination arm, 37 and 46.5 months, respectively. Development of grade 3 or 4 hypertension was correlated with two other polymorphisms *VEGF-A rs2010963* C/C (0%, *p* = 0.005) and *VEGF-A* rs3025039 T/T (8%, *p* = 0.022). Finally, a patient who developed grade 3 or 4 hypertension had a superior median OS of 36.7 months compared to 25.3 months in patients who did not develop hypertension (*p* = 0.012) [33]. Another prospective study, which tested the impact of *VEGF-A* gene polymorphisms on the pharmacodynamics of first-line bevacizumab-containing therapy in 137 breast cancer patients, pointed out the potential role of *VEGF-A* polymorphisms. *VEGF-A* rs2010963 C>T polymorphism revealed that patients bearing the T allele exhibited a longer time-to-progression (TTP) compared to homozygous for the C allele (11.5 vs. 9.7 months, *p* = 0.022). None of the other studied polymorphisms influence TTP, OS or clinical response. However, *VEGF-A* rs2010963 G>C was significantly associated with toxicity (*p* = 0.01) and patients with C allele were more prone to develop hypertension and thromboembolism [34] (Table 2).

Investigators of the randomized phase III GeparQuinto study tested whether SNPs in VEGF pathway genes correlate with complete pathological response after neoadjuvant treatment in 729 patients treated with bevacizumab and chemotherapy and in 725 treated only with chemotherapy for HER-2 negative breast cancer. Four SNPs of *VEGF-A* rs833058 (*p* = 0.003), rs699947 (*p* = 0.032), rs3025039 (*p* = 0.040), rs3025030 (*p* = 0.041), and one SNP of *VEGFR-1* (FLT1) rs79995976 (*p* = 0.049) were associated with better response, but they did not remain significant after correction for multiple testing. Investigators suggested that further research is warranted to clarify the predictive value of these markers [35] (Table 2).

*VEGFR-2* rs11133360 and *IL-8* rs4073 genotypes proved to be able to identify patients who will benefit more from bevacizumab treatment with a better outcome. In total, 113 mBC patients treated with first-line paclitaxel and bevacizumab were enrolled. The combination between specific *VEGFR-2* rs11133360 and *IL-8* rs4073 genotypes showed a statistically significant difference in median PFS for favorable and unfavorable genetic profiles (14.1 vs. 10.2 months, *p* < 0.0001) [36] (Table 2).

Genes not associated with VEGF pathway have also been investigated in mBC. Endothelial nitric oxide synthase (*eNOS)* rs1799983 polymorphisms and *IL-8* rs4073 T/A were also assessed by another group in 31 mBC patients treated with bevacizumab and chemotherapy. None of the analyzed polymorphisms were associated with RR. However, *IL-8* rs4073 A/A genotype showed a significantly lower PFS for the following combinations: T/T vs. A/A (13 vs. 8 months, *p* = 0.008), T/T vs. T/A vs. A/A (13 vs. 11 vs. 8 months, *p* = 0.02), T/T vs. T/A+A/A (13 vs. 11 months, *p* = 0.01), T/T+T/A vs. A/A (12 vs. 8 months, *p* = 0.01) and a lower OS, when compared with TT+TA genotype (26 vs. 51 months, *p* = 0.04). *eNOS* rs1799983 T/T genotype predisposed to statistically significant lower PFS compared to G/G genotype (11.5 vs. 26.5 months, *p* = 0.04), but no differences in OS. These results suggest that *IL-8* rs4073 T/A and *eNOS* rs1799983 G/T polymorphisms could be predictive of treatment outcomes [37] (Table 2).

Studies in mBC have identified promising genetic biomarkers, mainly polymorphisms of *VEGF-A* genes that are correlated with both efficacy and toxicity (Table 2).

### 3.2. Metastatic Colorectal Cancer (mCRC)

A recently published study investigated the effect of SNPs in VEGF-dependent and non-VEGF-dependent angiogenesis pathways on clinical outcomes in 46 mCRC patients treated with first-line bevacizumab-based treatment. Interestingly, two SNPs in *VEGF-A* and *ICAM-1* genes were identified as predictors of clinical outcomes. *VEGF-A* rs699947 A/A allele was significantly associated with increased PFS (32.6 vs. 8.1 months, *p* = 0.006) and OS (59.4 vs. 16.9 months, *p* = 0.043) compared to C/C allele. Besides, *ICAM-1* rs1799969 G/A allele was associated with prolonged OS (48.7 vs. 29.1 months, *p* = 0.036). Therefore, the authors concluded that *VEGF-A* and *ICAM-1* genes could be used to identify patients who will achieve long-term responses and benefit from bevacizumab-based therapies [38]. Mutations in *VEGF-A* rs699947 and *ICAM-1* rs1799969 have also been associated with better binding of bevacizumab to VEGF-A and lower bevacizumab clearance in patients with mCRC. These association may also partly explain the associations between these polymorphisms and favorable clinical outcomes [39]. Loupakis et al. conducted a retrospective exploratory analysis of *VEGF-A* polymorphisms. They used the genomic DNA of 111 mCRC patients treated with bevacizumab and FOLFIRI and as control 107 patients treated only with FOLFIRI. In the control group, no significant association was observed. However, in the bevacizumab group *VEGF-A* rs833061, C/C, C/T, and T/T polymorphisms were significantly associated with PFS (12.8 vs. 10.5 vs. 7.5 months, *p* = 0.0046) and OS (27.3 vs. 20.5 vs. 18.6 months, *p* = 0.0020). Based on the absence of differences in control, group investigators reported that the relation of *VEGF-A* rs833061 T/T genotype with shorter PFS was caused by the effect of bevacizumab (*p* = 0.011) [40]. Loupakis et al. also conducted a prospective study in a large clinically homogenous cohort of mCRC patients treated with first-line bevacizumab and FOLFIRI in order to evaluate candidate SNPs of VEGF/VEGFR pathway as potential predictors of response. They enrolled 424 patients and found a significant association between *VEGFR-2* rs12505758 C/T variants and PFS (*p* = 0.045). They also developed a multivariate model including histology, ECOG performance status, baseline LDH, number of metastatic sites, and primary tumor sites as covariates variants. It showed that patients with at least one C-allele exhibited lower PFS of 9.5 months compared with 10.9 months of homozygous for T/T (*p* = 0.012) [41] (Table 2).

Apart from *VEGF-A* polymorphisms Ulivi et al. also investigated polymorphisms of *eNOS* and their relation to response to bevacizumab in 237 mCRC patients (114 of them received chemotherapy and bevacizumab). Their panel included five SNPs for *VEGF-A* (rs1570360, rs699947, rs2010963, and rs833061), two for *eNOS*, as well as one variable number tandem repeat in intron 4 for *eNOS*. In the group of patients, who received bevacizumab *VEGF-A* rs2010963 T/T genotype was associated with a shorter median PFS compared to other genotypes (7.8 vs. 10.4 months, *p* = 0.036), while no significant difference was detected in the other groups. Similarly, *eNOS* rs1799983 G/T predisposed to shorter PFS (8.9 vs. 11.9 months, *p* = 0.013), whereas *eNOS* VNTR466 predisposed to longer PFS (10.9 vs. 9.1, *p* = 0.034). Interesting associations were also detected between OS and some polymorphism when bevacizumab was administrated. Patients had significantly shorter OS if *VEGF-A* rs2010963 T/T (8.6 vs. 22.7, *p* = 0.007) or *eNOS* rs1799983 G/T (20.1 vs. 26.1, *p* = 0.014) were present. On the other hand, *eNOS* VNTR 466 was also relevant to longer OS (24.8 vs. 20.6 months, *p* = 0.015). Thus, researchers concluded that a haplotype combination of *eNOS* polymorphisms is capable of identifying who may or will probably benefit from bevacizumab-based chemotherapy [42]. Gerger et al. published their results of studying a comprehensive panel of germline polymorphisms of angiogenesis genes and their ability to predict clinical outcome and tumor response. Their study population was 132 mCRC patients treated with first-line bevacizumab in combination with FOLFOX or XELOX. Increased OS was observed in patients carrying at least one G allele of epidermal growth factor gene (*EGF)* rs444903A>G (32.4 vs. 12.1 months, *p* = 0.012) and of insulin-like growth factor 1 gene (*IGF-1)* rs6220A>G (32.4 vs. 21.9). These results remained significant and in multivariate COX regression analysis. Another interesting finding was the association of the minor allele of *HIF-1a* rs11549465C>T with increased PFS compared to homozygous C/C (11.7 vs. 10.2, *p* = 0.038). Finally, in response rate significant differences were found between patients homozygous for the wild-type alleles of *CXCR1* rs2234671G>C compared to those carrying one or two minor alleles (71% vs. 37% vs. 17%, *p* < 0.001) and of *VEGFR-2* rs2305948C>T (57% vs. 29% vs. 13%, *p* = 0.024). In contrast, lower response rate was presented in patients harboring wild-type of *CXCR2* rs2230054T>C compared to heterozygous and homozygous for one minor allele (38% vs. 56% vs. 79%, *p* = 0.008) and of *VEGFR* rs2227983G>A (43% vs. 55% vs. 82%, *p* = 0.024) [43]. In 40 mCRC patients treated with bevacizumab and FOLFIRI germline *VEGF-A* genes polymorphisms were associated with treatment outcomes. PFS was shorter in carriers of *VEGF-A* rs833061 G/G compared to G/A +A/A (8.9 vs. 15.4 months, *p* = 0.007) and *VEGF-A* rs1570360 G/G compared to G/A +A/A (9.8 vs. 16 months, *p* = 0.03). They also performed multivariate analysis, including biochemical variables known to influence prognosis, which showed that *VEGF-A* rs1570360 G/G retained an independent predictive value for PFS compared to G/A + A/A (*p* = 0.02). Concerning response rate, *VEGF-A* rs2010963 G/G was significantly associated with response compared to G/C + C/C (64% vs. 14%, *p* = 0.03) [44]. Another promising predictive marker is CD133. In a study of 91 mCRC or recurrent CRC patients treated with first-line bevacizumab and FOLFOX or XELOX, those with high intratumoral gene expression levels of CD133 (>7.76) had significantly better tumor response compared to those with gene expression ≤7.76 (86% vs. 38%, *p* = 0.003). Moreover, these patients presented higher VEGF-A or VEGFR expression levels (VEGF-A, VEGFR-2, VEGFR-3, *p* < 0.09, and VEGFR-1, *p* < 0.01). In combination analysis after adjustment for sex and number of metastatic sites, multivariate analysis showed that CD133 polymorphisms of rs2286455 and rs3130 are independent prognostic factors for PFS (*p* = 0.002) [45]. The same conclusion was drawn by another study in an mCRC setting, where the authors stated that their results should be validated prospectively, in larger cohorts. In total, 173 patients treated with bevacizumab in combination with FOLFIRI or XELIRI participated and their blood samples genotyped for SNPs *VEGF-A* (rs1570360, rs699947, and rs2010963). *VEGF-A* rs1570360 G/G was more frequent in non-responders compared with responders (65.5% vs. 39.8%, *p* = 0.032). Furthermore, it was associated with an inferior median OS compared with GA (*p* = 0.016) or with GA and AA combination (*p* = 0.017). In multivariate analysis, the *VEGF-A* rs1570360 G/G genotype remained a significant adverse factor for OS [46]. An Asian group conducted a study to evaluate 12 SNPs of angiogenic genes in 125 mCRC patients, who received first-line bevacizumab and chemotherapy. They reported that *VEGF-A* rs833061T/T was associated with superior RR compared to its alternative genotypes (75.9 vs. 50.8%, *p* = 0.008). Median PFS (8.7 vs. 6.6 months, *p* = 0.001) and OS (26.4 vs. 16.1 months, *p* = 0.038) were superior in patients with the fms-related tyrosine kinase 1 (*FLT1*) rs9513070A/A genotype. Haplotype analysis associated the *FLT1* rs9513070/rs9554320/rs9582036 G/C/A haplotype with inferior PFS (*p* = 0.004) and OS (*p* = 0.041) [47]. Finally, a meta-analysis including 60 studies and analyzing five *VEGF-A* polymorphisms (rs3025039, rs833058, rs1570360, rs699947, and rs2010963), identified a significant prognostic relationship of *VEGF-A* rs833058 variants, as the carriers of them showed a highly statistically significant improvement in OS (*p* = 0.004) [48] (Table 2).

Independently of angiogenesis genes, Matsusaka et al. investigated if SNPs in genes involved in IL-6/STAT3 signaling, *IL-6* (rs2069837 and rs1800795) and *STAT3* (rs744166 and rs4796793) can predict PFS, OS, and response to first-line FOLFIRI plus bevacizumab in mCRC setting. Researchers used data from two randomized phase III trials: TRIBE (223 patients), training cohort, and FIRE-3 (288 patients, validation cohort, and 264 control cohort). *IL-6* rs2069837G was associated with longer PFS than the A/A genotype both in TRIBE (9.4 vs. 11.1 months, *p* = 0.004) and FIRE-3 (8.8 vs. 10.9 months, *p* = 0.015) trials. These associations were confirmed in multivariable analyses and were not seen in the control cohort [49]. A study conducted in 58 patients treated with bevacizumab, irinotecan, and fluoropyrimidines for mCRC evaluated the association between *CD133* rs3130 and rs2286455 polymorphisms and treatment outcomes. Significant association was observed only for rs3130 C/C genotype, which was associated with reduced toxicity of treatments (*p* = 0.0017) and with lower OS (*p* = 0.002) [50]. Matsusaka et al. again studied an important mechanism of resistance to angiogenesis inhibition: the ability of epithelial-mesenchymal transition (EMT) pathway genetic variants to predict the efficacy of antiangiogenic therapy. They analyzed associations between functional SNPs in EMT-related genes and outcomes of first-line bevacizumab-based chemotherapy in 143 mCRC patients; they also used a control of 77 patients treated with cetuximab-based chemotherapy. Studied SNPs included *TWIST1* (rs2285682 and rs2285681), *ZEB1* (rs10826943 and rs2839658), *SNAIL* (rs1543442 and rs4647958), and *E-cadherin* (rs16260). Carriers of a *TWIST1* rs2285682 G allele had a significantly longer median PFS compared with those with the T/T genotype (18.1 vs. 13.3 months, *p* = 0.003) and OS (44.1 vs. 29.2 months, *p* = 0.001) in the bevacizumab cohort. These associations remained significant also in multivariate analysis. Moreover, improved PFS and OS were associated in multivariate analysis revealed with the combination of female sex and *TWIST1* rs2285682 G (PFS, *p* = 0.007; OS, *p* = 0.001) and rs2285681 G genotypes (PFS, *p* < 0.001; OS, *p* < 0.001). No significant associations were found in the cetuximab cohort, suggesting that *TWIST1* polymorphisms may serve as clinically useful biomarkers for antiangiogenic therapy [51] (Table 2).

Similarly, to mBC studies, polymorphisms of *VEGF-A, eNOS*, and *VEGF-R* genes were associated significantly with outcomes in mCRC patients treated with bevacizumab (Table 2).

### 3.3. Lung Cancer

Apart from circulating biomarkers ABIGAIL trial also performed exploratory analyses of 12 SNPs across three genes are of *VEGF-A, VEGFR-1*, and *VEGFR-2*. Genetic variants at *VEGF-A* and *VEGFR-1* were associated with treatment outcome. Especially, *VEGF* rs699947 C>A was associated with >50% higher response rate, rs2010963 C>T was associated with higher incidence of hypertension and *VEGFR-1* rs9554316 G/T was associated with >30% higher risk of progression and >40% higher risk of death [52] (Table 2).

In line with mBC and mCRC studies *VEGF-A* and *VEGF-R* genes were identified as potential biomarkers (Table 2).

### 3.4. Ovarian Cancer

Schultheis AM et al. used genomic DNA from blood samples of 53 patients with recurrent or metastatic epithelial ovarian cancer and evaluated the association between angiogenesis gene polymorphisms and outcome of bevacizumab and low-dose cyclophosphamide treatment. They found that patients homozygous for *CXCR2* rs2230054 T/T polymorphism had significantly lower median PFS compared to patients carrying at least one C allele (3.7 vs. 7.4 months, *p* = 0.026). Patients heterozygous for *VEGF-A* rs3025039 C/T had longer median PFS of 11.8 months compared to 5.5 months for homozygous C/C and only 3.2 months for homozygous T/T. When they combined genetic data, they found that patients carrying both adremmedullin 3′ and alleles <14CA repeats had lower median PFS compared to those with at least one allele ≥14CA (3.4 vs. 6.4 months) or both alleles ≥14CA (3.4 vs. 7.2 months) (*p* = 0.008). Additionally, patients with A/A or A/T genotype at the −251 locus of the IL-8 gene had lower response rate than those who were T/T homozygous (*p* = 0.006). These data suggest that genes independent of VEGF play an important role in the mechanism of resistance to anti-VEGF therapy [53] (Table 2).

Similarly, to mBC and mCRC studies *VEGF-A* and *IL-8* genes were identified as potential biomarkers (Table 2).

### 3.5. Renal-Cell Cancer (RCC)

One large study investigated DNA from patients from two phase III randomized studies (AViTA and AVOREN). Correlation of 138 SNPs in the VEGF pathway with PFS and OS was assessed. *VEGFR-1* rs9582036 AA, was significantly associated with longer OS (*p* = 0.00014) and PFS (*p* = 0.00081) in the bevacizumab group of AViTA after correction for multiplicity, but no effects in placebo patients (*p* = 0.041) were seen or recorded, indicating that the *VEGFR-1* locus containing this SNP serves as a predictive marker for bevacizumab treatment outcome. Interestingly, this locus correlated significantly with PFS (*p* = 0.033) also in the bevacizumab group in AVOREN in patients with metastatic pancreatic adenocarcinoma [54] (Table 2).

### 3.6. Glioblastoma

The correlation between 14 SNPs of *VEGF-A, VEGFR-2*, and *HIF-1a* with treatment efficacy and toxicity was examined in 54 patients receiving bevacizumab and sorafenib for recurrent glioblastoma. Mutations of *VEGF-A* and *VEGFR-2* promoters were significantly associated with 6-month PFS success. It was increased for mutant *VEG*F-A rs699947 (*p* = 0.011), rs833061 (*p* = 0.013), and heterozygous *VEGFR-2* rs2071559 (*p* = 0.025), but it was decreased for mutant *VEGF-A* rs1005230 (*p* = 0.011) and rs1570360 (*p* = 0.004). Notably, increased incidence of grade≥3 hypertension was observed in heterozygous patients for *VEGF-A* promoter rs1005230 (*p* = 0.006), rs699947 (*p* = 0.006), and rs833061 (*p* = 0.010). Researchers concluded that their data represent the first evidence that *VEGF-A* and *VEGFR-2* genetic polymorphisms could predict outcome in glioblastoma patients treated with bevacizumab [55]. These findings are in line with the results of studies in other indications (Table 2).

Most of the studies revealed a significant correlation between specific polymorphisms and genotypes with the response, OS, and PFS. It is also important that large, prospective studies and meta-analyses have found a positive relation between genetic factors and outcome. In addition, the association has been confirmed in many cases by multivariate analyses. The most promising polymorphisms are those of *VEGF-A, ICAM-1, VEGF-R, eNOS,* and *IL-8* (Table 2). Further randomized trials and genome-wide association studies in the future could offer solid and validated results that will make antiangiogenic treatment more precise.

## 4. Bevacizumab Levels-Therapeutic Drug Monitoring (TDM)

As already mentioned above an overall benefit of bevacizumab in the treatment of different tumors has been established. However, clinical outcomes can be highly variable, with some patients responding remarkably well, while others not. Another important parameter is side-effects such as hypertension, bleeding, phlebitis, embolism, and wound healing problems that often lead to delay or even permanent discontinuation of the treatment [56]. Thus, due to this heterogeneous response, the real clinical impact of bevacizumab remains unclear. A characteristic example is that it delays the progression of brain and breast cancers, it does not improve overall survival [57,58]. Pharmacokinetic parameters are among of the most important parameters, which influence drug action and clinical response. Monoclonal antibodies present very variable and complicated pharmacokinetics [59,60]. Thus, modulation of the pharmacokinetic parameters of bevacizumab could explain the inter-individual variability observed in patients. The mean half-life of bevacizumab is nearly 20 days [13], however, large individual differences were noted, with a range between 11 and 50 days. Presumably, this inter-individual variability could in some degree explain variable responses to the treatment. For example, when treatment is administered every two weeks, a patient for whom the half-life is 50 days would present an excess of circulating antibody from the second dose. This could result in side effects during therapy. In contrast, a patient for whom the half-life is only 11 days will rapidly clear bevacizumab, and this could impede treatment efficacy. Although these differences are remarkable and could impede treatment efficacy, a standardized administration protocol is recommended instead of a personalized treatment regimen. In order to implement more precise and personalized regimens based on bevacizumab pharmacokinetics and to develop therapeutic drug monitoring (TDM)-guided protocols, validated, fast and relatively cheap analytical techniques are needed.

In a non-oncology setting, TDM is a standard-of-care and cost-effective practice for several classes of antibiotics, immunosuppressants, antiepileptics, HIV agents, and many other drugs in clinical practice [61]. Moreover, the TDM-guided approach has been proved to improve safety and efficacy of both classic cytotoxic drugs as well as targeted therapies with small molecules tyrosine kinase inhibitors [62,63,64,65,66,67,68]. Similarly, there are some studies that support the TDM-approach for the treatment with monoclonal antibodies such as rituximab and cetuximab [69,70,71]. The progress in the field for bevacizumab includes the development of a validated ELISA method for levels measurements [72].

A recently published study assessed exposure-survival relationships in 46 mCRC patients receiving bevacizumab in combination with chemotherapy as first-line treatment. They found a strong positive correlation between bevacizumab trough levels and survival (*p* = 0.0004). Furthermore, three distinct groups of exposure were identified. The low (≤41.9mg/L) and medium (43–87.2 mg/L) groups with median OS of 12.8 and 36 months, respectively (*p* = 0.0003); and high group (≥87.9 mg/L), where the OS was not met as majority of patients were alive 60 months after the initiation of treatment. These findings suggest that bevacizumab trough concentration could be used both as a predictive biomarker of OS and as a tool to optimize treatment [73]. Nugue G et al. also analyzed bevacizumab concentrations in 17 breast cancer and glioblastoma patients throughout the first quarter of treatment. All of the patients were treated with bevacizumab 10 mg/kg every two weeks and blood samples were collected just before bevacizumab infusion. Average blood concentrations were 88 mg/L (54–149 mg/L) and following the first dose and 213 mg/L (73–411 mg/L) after the sixth dose administered. However, the individual values were scattered, with a mean four-fold difference between the lowest and the highest concentration for each dose administered. For the pharmacodynamic assessment, only 13 glioblastoma patients were used and breast cancer patients were excluded. Investigators classified patients in three groups according to clinical data: patients with side effects, non-responders, and good responders. Low serum bevacizumab concentrations were associated with a lack of efficacy, while high concentrations were associated with side effects. Thus, they concluded that serum bevacizumab concentration appears to be a useful clinical pharmacodynamic marker [74]. In 2016 two other groups published their results regarding the role of bevacizumab plasma concentrations. Firstly, in 20 mCRC receiving bevacizumab and chemotherapy bevacizumab, not bevacizumab-bound VEGF, total VEGF, and Ang-2 plasma concentrations were determined. Blood collection was performed at baseline and immediately before the second and ifth cycles. Bevacizumab levels were quite similar immediately before the second cycle between all patients, measuring 2.61 ± 1.10, 5.51 ± 5.28 and 2.33 ± 0.94 μg/mL in partial response (PR), stable disease (SD), and progression of disease (PD), respectively. However, drug levels change before the fifth cycle was different among patients with PR compared to those with SD and PD with a 0.72 ± 0.25 and 2.10 ± 0.13-fold increase, respectively. Moreover, assessment of VEGF as total and not bound before the second and fifth cycles revealed a significant decrease in the ratio of not bound to bevacizumab VEGF to total VEGF before the second and fifth cycles for patients who respond to treatment (*p* < 0.05) from 26.65% to 15.5% in PR group and from 53.41% to 34.95% in SD group. On the other hand, patients who showed a PD presented a significantly higher ratio (*p* < 0.05) before the second cycle, 51.71% vs. 25.99% [75]. A study by Caulet M et al. aimed to quantify individual factors affecting bevacizumab pharmacokinetics and assess the relationship between bevacizumab concentrations and clinical outcomes, concluding that decreased OS and PFS are associated with high bevacizumab concentration. In total, 137 mCRC patients treated with bevacizumab and chemotherapy were included in the trial. Pharmacokinetics were assessed by using a two-compartment pharmacokinetic population model, OS, and PFS by using Cox models. According to the results, the volume of bevacizumab distribution was significantly increased with height (*p* < 0.0001) and was higher in patients carrying a 3/3 variable number tandem repeat of the FCGRT gene (*p* = 0.039). Higher elimination rate was observed with increased baseline carcinoembryonic antigen (CEA) (*p* = 0.00029) and VEGF (*p* = 0.011) concentrations and was higher in patients with extra-hepatic metastases (*p* = 0.014). Moreover, the multivariate analysis showed that only baseline VEGF concentration and trough bevacizumab concentrations were independent risk factors for progression. Median PFS was 10.2 months and significant shorter when trough concentrations were <15.5 mg/L compared to through concentrations ≥15.5 mg/L (8.7 vs. 13.6 months) [76].

## 5. Conclusions

As described above, several proteinic biomarkers and gene polymorphisms could be correlated with treatment outcomes. It is crucial to assess the replication of findings and focus on the significant findings identified by multiple studies in order to achieve better control of the false discovery rate. However, differences in tumor biology should be taken into account to interpret differences in results from different tumor types. Therefore, VEGF-A levels and *VEGF-A* rs699947 and rs1570360 are the most promising proteinic and genomic biomarkers, respectively, as they have been correlated with favorable clinical outcomes in several studies. Circulating VEGF-A levels could also be measured from a simple blood sample, and there are commercially available, standardized, validated ELISA kits, which can be easily added and applied at the point of care laboratories. Another fascinating and significant signal is the association of exposure to bevacizumab and survival, which could be used in order to develop TDM approaches.

The available data should be combined in order to optimize the treatment planning for each individual. As a result, a TDM and genetic biomarker-guided dose strategy appears feasible in the near future. In conclusion, barriers exist to the implementation of a personalized approach in bevacizumab treatment, but in the near future, this may feasibly improve quality of care and treatment outcomes.

## Figures and Tables

**Table 1:** Available studies on proteinic biomarkers associated with response to bevacizumab.

Reference	Tumor Type	Number of Patients	Treatment	Biomarkers	Findings
Alvarez Secord A et al. [30]	Ovarian cancer	751	Chemotherapy (carboplatin and paclitaxel) plus placebo vs. chemotherapy (carboplatin and paclitaxel) plus bevacizumab	Plasma levels of IL-6, Ang-2, OPN, SDF-1, VEGF-D, IL-6 R, and GP130 at baseline	High IL-6 (>22.1 pg/mL) levels were associated with significantly improved PFS and OS in patients treated with bevacizumab
Burstein HJ, et al. [17]	mBC	56	Vinorelbine plus bevacizumab	Plasma VEGF-A levels at baseline	Baseline levels >32.6 pg/mL associated with shorter time to progression and no tumor control
Dowlati A et al. [27]	NSCLC	878	Carboplatin and paclitaxel vs. carboplatin and paclitaxel plus bevacizumab	Plasma levels of VEGF-A, bFGF, soluble ICAM-1, and E-selectin at baseline and after cycle 2.	High baseline VEGF-A plasma levels (>35.7 pg/mL) were associated with better response in patients treated with bevacizumab. Whereas, low ICAM-1 levels (≤260.5 ng/mL) were prognostic for survival and predictive of response regardless of the administration of bevacizumab
Horn L et al. [29]	Small-cell lung cancer	63	Cisplatin and etoposide plus bevacizumab	Plasma levels of VEGF-A, soluble VCAM, ICAM-1, E-selectin and bFGF at baseline and after completion of cycle 2	High baseline VCAM plasma levels were associated with higher risk of progression and death
Jubb AM, et al. [18]	mBC	462	Capecatibine vs. capecatibine plus bevacizumab	Expression of VEGF-A, VEGF-B, THBS-2, Flt4, VEGF-C, PDGF-C, neuropilin-1, delta-like ligand D114, Bv8, p53, and thymidine phosphorylase in tumor tissue	VEGF-A expression was predictive of PFS in patients treated with bevacizumab
Jubb AM, et al. [19]	mCRC	813	IFL vs. IFL plus bevacizumab	Expression of the stromal and epithelial VEGF-A, THBS-2 score, MVD in tumor tissue	None of the tested biomarkers were predictive of survival
Jürgensmeier, J, et al. [20]	mCRC	1254	mFOLFOX6 plus cediranib vs. mFOLFOX6 plus bevacizumab	Plasma VEGF-A, soluble VEGFR-2 and CEA levels at baseline	High baseline VEGF-A (>98 pg/mL) and CEA levels were associated with shorter PFS and OS regardless of treatment
Koeptz S et al. [24]	mCRC	43	FOLFIRI plus bevacizumab	37 plasma cytokines and angiogenic factors (CAFs)	Significant elevation of pro-angiogenic cytokines (bFGF, PIGF, and hepatocytes growth factor) was associated with resistance to treatment
Mok T et al. [28]	NSCLC	303	Chemotherapy (carboplatin and paclitaxel, carboplatin and gemcitabine) plus bevacizumab	Plasma levels of VEGF-A, VEGFR-1, VEGFR-2, bFGF, E-selectin, ICAM-1, PIGF at baseline and through treatment	Low baseline VEGF-A plasma levels were correlated with prolonged PFS and OS
Nixon A et al. [31]	Renal-cell cancer	424	interferon-A plus bevacizumab vs. interferon-A	Baseline plasma levels of 32 factors related to tumor growth, angiogenesis, and inflammation	IL-6 and HGF plasma levels at baseline were positively correlated with OS in patients treated with bevacizumab
Rozoni M et al. [25]	mCRC	40	Chemotherapy (FOLFIRI, FOLFOX4, XELOX, FOLFOXIRI) plus bevacizumab	Absolute number of total circulating endothelial cells (tCECs), resting CECs (rCECs) and endothelial progenitor cells (CEPs) at baseline and before the administration of the third and sixth course of treatment and compared their results with a control group of 50 healthy volunteers	The absolute number of tCEC and rCEC <40 cells/mL at baseline was associated with longer PFS
Sathornsumetee S et al. [32]	Malignant gliomas	45	Irinotecan plus bevacizumab	Tumor expression of VEGF-A, VEGFR-2, CD31, CA9, and HIF-2a	High VEGF-A expression (>5000 pixels/x400 field) was associated with increased likelihood of radiographic response and high CA9 expression (>10000 pixels/high-powered field) was associated with poor 1-year survival
Tsai HL, et al. [21]	mCRC	57	FOLFIRI plus bevacizumab	Pre-and post-therapeutic VEGF-A IHC expression	Low post-therapeutic VEGF-A scores (0 and 1) and decreased peri-therapeutic VEGF-A scores were significant predictive factors of response. High pre-therapeutic VEGF-A scores (2 and 3) were not a significant predictive factor, but a decrease from score 2 to 1 or 0 between pre-treatment and post-treatment period was a significant predictor factor of response to bevacizumab. Decreased peri-therapeutic VEGF-A scores were also significantly associated with higher 6-month PFS
Willett CG, et al. [22]	Rectal cancer	32	Neoadjuvant bevacizumab monotherapy, bevacizumab plus fluorouracil and radiotherapy	PIGF, IL-6, CECs, sVEGF-A1, and VEGFR at baseline, 3 and 12 days after a dose of bevacizumab monotherapy, 32 days after initiation of neoadjuvant bevacizumab, fluorouracil and radiotherapy and 1 week before surgery	Greater than 2-fold increases in plasma PIGF after bevacizumab monotherapy was associated with minimal disease at surgery. Pretreatment sVEGFR1 inversely correlated with the extent of regression, and early kinetics of PlGF after bevacizumab, and VEGF-A and IL-6 levels during combined treatment may predict better outcomes
Yamamoto Y et al. [26]	mCRC	99	Chemotherapy (mFOLFOX6, XELOX) plus bevacizumab	Baseline plasma levels of ICAM-1 and IL-8	Plasma levels of ICAM-1 and IL-8 at baseline were significantly associated with shorter PFS and shorter PFS and OS respectively

**Table 2:** Available studies on gene polymorphisms associated with response to bevacizumab.

Reference	Tumor Type	Number of Patients	Treatment	Biomarkers	Findings
Allegrini G et al. [36]	mBC	113	Paclitaxel plus bevacizumab	SNPs of *VEGF-A, VEGFR-2, IL-8, HIF-1α, EPAS-1* and *TSP-1*	*VEGFR-2* rs11133360 and *IL-8* rs4073 genotypes were significantly associated with PFS
Aravantinos G et al. [50]	mCRC	58	Irinotecan and fluoropyrimidines plus bevacizumab	*CD133* rs3130 and rs2286455 polymorphisms	*CD133* rs3130 C/C genotype was associated with shorter OS
Di Salvatore M et al. [37]	mBC	31	Paclitaxel plus bevacizumab vs. paclitaxel and carboplatin plus bevacizumab	*eNOS-894* G/T, *eNOS-*786 T/C, *COX2-8473* C/T, IL-*8 251* T/A polymorphisms	*IL-8* rs4073 T/A and *eNOS* rs1799983 G/T polymorphisms were associated with improved PFS, and PFS and OS respectively
Etienne-Grimaldi MC et al. [34]	mBC	137	Chemotherapy (paclitaxel, docetaxel, vinorelbine, taxane combination, non-taxane combination) plus bevacizumab	*VEGF-A* polymorphisms in blood samples	*VEGF-A* rs2010963 C>T polymorphism was associated with longer time-to-progression
Formica V et al. [44]	mCRC	40	FOLFIRI plus bevacizumab	*VEGF-A* gene polymorphisms	*VEGF-A* rs833061 G/G and rs1570360 G/G were associated with shorter PFS, *VEGF-A* rs2010963 G/G was associated with higher response rates
Galanis E et al. [55]	Glioblastoma	54	Sorafenib plus bevacizumab	*VEGF-A, VEGFR-2* and *HIF-1a* genes polymorphisms	Mutant *VEG*F-A rs699947, rs833061 and heterozygous *VEGFR-2* rs2071559 were associated with increased 6-month PFS success, but mutant *VEGF-A* rs1005230 and rs1570360 were associated with decreased 6-month PFS success
Gerger A et al. [43]	mCRC	132	Chemotherapy (FOLFOX, XELOX) plus bevacizumab	Comprehensive panel of VEGF-dependent and VEGF-independent angiogenesis genes polymorphisms	*EGF* rs444903 A>G and *IGF-1* rs6220 A>G were associated with increased OS, *CXCR1* rs2234671 G>C was associated with higher response rates
Hein A et al. [35]	mBC	1454	Neoadjuvant epirubicin and cyclophosphamide followed by docetaxel vs. epirubicin and cyclophosphamide followed by docetaxel plus bevacizumab	SNPs in VEGF pathway genes in blood samples	*VEGF-A* rs833058, rs699947, rs3025039, rs3025030 and *VEGFR-1* rs79995976 were associated with higher rates of pathological complete response, but they did not remain significant after correction for multiple testing
Koutras A et al. [46]	mCRC	173	Chemotherapy (FOLFIRI, XELIRI) plus bevacizumab	SNPs of *VEGF-A* (rs1570360, rs699947, rs2010963)	*VEGF-A* rs1570360 G/G was associated with no response to treatment and inferior OS
Lambrechts D et al. [54]	Renal-cell cancer	649	Interferon alfa-2a plus bevacizumab vs. interferon alfa-2a plus placebo	Comprehensive panel of VEGF-dependent angiogenesis genes polymorphisms	*VEGFR-1* rs9582036 AA was associated with longer OS and PFS in patients treated with bevacizumab
Loupakis F et al. [40]	mCRC	218	FOLFIRI vs. FOLFIRI plus bevacizumab	*VEGF-A* polymorphisms	*VEGF-A* rs833061 C/T polymorphism was significantly associated with PFS and OS in patients treated with bevacizumab
Loupakis F et al. [41]	mCRC	424	FOLFIRI plus bevacizumab	SNPs of VEGF/VEGFR pathway	*VEGFR-2* rs12505758 T/T was associated with improved PFS
Matsusaka S et al. [49]	mCRC	511	FOLFIRI plus bevacizumab	*IL-6* (rs2069837, rs1800795) and *STAT3* (rs744166, rs4796793) genes polymorphisms	*IL-6* rs2069837G was associated with longer PFS
Matsusaka S et al. [51]	mCRC	143	Chemotherapy (FOLFOX, XELOX) plus bevacizumab	SNPs in *TWIST1* (rs2285682, rs2285681), *ZEB1* (rs10826943, rs2839658), *SNAIL* (rs1543442, rs4647958), and *E-cadherin* (rs16260)	*TWIST1* rs2285682 G allele was associated with longer PFS and OS in patients treated with bevacizumab
Pallaud C et al. [52]	NSCLC	303	Chemotherapy (carboplatin plus paclitaxel or carboplatin plus gemcitabine) plus bevacizumab	*VEGF-A, VEGFR-1* and *VEGFR-2* polymorphisms	*VEGF-A* rs699947 C>A was associated with higher response rate, rs2010963 C>T was associated with higher incidence of hypertension and *VEGFR-1* rs9554316 G/T was associated with higher risk of progression and death
Papachristos A et al. [38]	mCRC	46	Chemotherapy (mFOLFOX6, FOLFIRI, CapOX, CapIRI) plus bevacizumab	*VEGF-A* (rs2010963, rs1570360, rs699947) and *ICAM-1*(rs1799969, rs5498) polymorphisms in blood and KRAS, NRAS, BRAF mutations in tumor	*VEGF-A* rs699947 A/A was associated with increased PFS and OS, whereas *ICAM-1* rs1799969 G/A and *BRAF* wild-type were associated with prolonged OS
Pohl A et al. [45]	mCRC	91	Chemotherapy (FOLFOX, XELOX) plus bevacizumab	Intratumoral gene expression levels of CD133, *VEGF-A* and *VEGFR* genes	High gene expression of CD133 (>7.76) was associated with higher response rates and longer PFS
Schneider BP et al. [33]	mBC	363	Paclitaxel vs. paclitaxel plus bevacizumab	*VEGF-A* and *VEGFR-2* polymorphisms in tumor block samples	*VEGF-A* rs699947 and rs1570360 A/A were associated with significantly longer OS in patients treated with bevacizumab
Schultheis, AM et al. [53]	Ovarian cancer	53	Oral cyclophosphamide plus bevacizumab	Comprehensive panel of VEGF-dependent and VEGF-independent angiogenesis genes polymorphisms	*CXCR2* rs2230054 T/T was associated with lower PFS, *VEGF-A* rs3025039 was associated with longer PFS, and *IL-8* A-251T was associated with higher response rates
Sohn BS et al. [47]	mCRC	125	Chemotherapy (mFOLFOX6, FOLFIRI, CapOX, CapIRI) plus bevacizumab	Polymorphisms in *VEGF-A, FLT1, KDR, IL-8, IL-10, CXCR2* and *COL18A1* genes	*VEGF-A* rs833061 T/T was associated with higher response rates and *FLT1* rs9513070 A/A genotype was associated with longer OS
Ulivi P et al. [42]	mCRC	237	Chemotherapy (FOLFOX4, FOLFIRI) vs. chemotherapy (FOLFOX4, FOLFIRI) plus bevacizumab	*VEGF-A* (rs1570360, rs699947, rs2010963, rs833061) and *eNOS* (rs1799983 VNTR466) polymorphisms	*VEGF-A* rs2010963 T/T and *eNOS* rs1799983 G/T were associated shorter PFS and OS, whereas *eNOS* VNTR466 predisposed to longer PFS and OS in patients treated with bevacizumab
